# Clinical profile and outcome of patients with chronic inflammatory arthritis and metabolic syndrome

**DOI:** 10.1007/s11739-020-02520-y

**Published:** 2020-10-20

**Authors:** Giovanni Cioffi, Ombretta Viapiana, Luigi Tarantini, Giovanni Orsolini, Luca Idolazzi, Federica Ognibeni Sonographer, Andrea Dalbeni, Davide Gatti, Angelo Fassio, Maurizio Rossini, Alessandro Giollo

**Affiliations:** 1 Rheumatology Section, Department of Medicine, University of Verona, Verona, Italy; 2 Department of Cardiology, Ospedale Civile S. Martino, Belluno, Italy; 3 Department of Medicine, General Medicine and Hypertension Unit, University of Verona and Azienda Ospedaliera Universitaria Integrata of Verona, Verona, Italy

**Keywords:** Metabolic syndrome, Rheumatoid arthritis, Psoriatic arthritis, Ankylosing spondylitis, Clinical outcomes, Cardiovascular risk factors, Cancer, Prognosis

## Abstract

Systemic chronic inflammation may favor the onset of metabolic syndrome (MetS) which represents a risk factor for CV events. Rheumatoid arthritis (RA), ankylosing spondylitis (AS) and psoriatic arthritis (PsA) are disorders with high prevalence of MetS. We assessed the factors associated with MetS and its prognostic role in non-selected RA/AS/PsA patients. Between March 2014 and April 2016, 458 patients (228 RA, 134 PsA, 96 AS) selected for a primary prevention program for cardiovascular diseases were analyzed. Primary and co-primary end points were a composite of all-cause death/all-cause hospitalization and CV death/CV hospitalization, respectively. MetS was diagnosed according to the IDF Task Force on Epidemiology and Prevention. Patients were divided into MetS + (73 = 16%) and MetS − (385 = 84%). At multivariate logistic analysis, cancer, moderate/high disease activity, higher LV mass (LVM) and degree of LV diastolic dysfunction were independently associated with MetS. At 36-month follow-up, the event rate for primary/co-primary end point was 52/15% in MetS + vs 23/7% in MetS − (both *p* < 0.001). At multivariate Cox regression analysis, MetS was related to primary end point (HR 1.52 [CI 1.01–2.47], *p* = 0.04) together with higher LVM, disease duration and higher prevalence of biologic DMARDs refractoriness, and to co-primary end point (HR 2.05 [CI 1.16–3.60], *p* = 0.01) together with older age and higher LVM. The RA/AS/PsA phenotype MetS + is a subject with moderate/high disease activity, LV structural and functional abnormalities at increased risk for cancer. MetS + identifies RA/AS/PsA patients at higher risk for CV and non-CV events, independently of traditional CV risk factors analyzed individually and traditional indexes of inflammation.

## Introduction

Metabolic syndrome (MetS) represents a cluster of cardio-metabolic disorders including obesity and visceral adiposity, insulin resistance, dyslipidemia, hyperglycemia and hypertension. Scientific evidence has eliminated legitimate doubts about the association between MetS and adverse prognosis in a number of clinical settings, with special emphasis on those with high prevalence and public health impact such as cardiovascular (CV) morbidity and mortality [1–5]. However, there is still debate in the scientific community about whether MetS facilities the prediction of adverse clinical events beyond use of single risk factors [6–8], also in relation to the different definitions of MetS itself [9, 10]. It has been clearly documented that MetS is associated with more severe left ventricular (LV) hypertrophy and other manifestations of preclinical CV disease [11, 12], while conflicting results exist about its influence on LV systolic function [13, 14]. Collectively, available data from the literature suggest that the CV risk predicted by MetS might be mediated, at least in part, by changes in LV geometry, diastolic function [15] and increased aortic stiffness [16, 17]. Systemic chronic inflammation and the increased production of pro-inflammatory cytokines may favor the onset of MetS [18–21]. This is the reason why patients with chronic inflammatory arthritis such as rheumatoid arthritis (RA), psoriatic arthritis (PsA) and ankylosing spondylitis (AS) have increased prevalence of MetS [22–24]. Furthermore, in these patients, the activation of pro-inflammatory signaling pathways stimulates several biological markers of inflammation contributing to CV disease. Thus, MetS and altered secretion patterns of pro-inflammatory molecules could be the link between chronic inflammatory arthritis and CV diseases. Although intuitive and rational, this association has never been demonstrated in the setting of patients with chronic inflammatory arthritis. Furthermore, it is still uncertain whether MetS represents an adverse risk factor for the occurrence of adverse clinical events in patients with chronic inflammatory arthritis such as RA/AS/PsA. Accordingly, this study aimed to assess the prevalence and factors related to MetS in patients suffering from RA/AS/PsA, and to evaluate whether MetS is associated with more incident CV and/or non-CV events, independent of the traditional CV risk factors analyzed individually (including LV hypertrophy) and of traditional markers of inflammation and disease activity.

## Materials and methods

### Study population

The design of the study was prospective. The study population comprised non-institutionalized subjects > 18 years of age in stable sinus rhythm with RA diagnosed according to the 2010 ACR/EULAR classification criteria [25], PsA and AS diagnosed by the CASPAR and the ASAS criteria as recently summarized by Rudwaleit and Taylor [26]. Participants were consecutively recruited from March 2014 to April 2016 at the Division of Rheumatology, Department of Medicine, University and Azienda Ospedaliera Universitaria Integrata of Verona (Italy). They underwent clinical, laboratory and echocardiographic evaluations as part of a primary prevention program for CV diseases. Exclusion criteria were the presence of symptoms/signs of cardiac disease, a history of myocardial infarction, myocarditis or heart failure, coronary heart disease diagnosed by clinical, electrocardiographic evaluation at rest and by the results of exercise/scintigraphy/echo-stress test, alcoholic or primary hypertrophic cardiomyopathy, prior myocardial revascularization, significant valve heart disease and atrial fibrillation. All patients gave written informed consent signing a specific institutional consent form; the study was approved by Ethical Committees of the Verona University and conforms to the ethical guidelines of the Declaration of Helsinki as revised in 2000.

### Definitions

Metabolic syndrome was diagnosed according to the joint interim statement of the International Diabetes Federation Task Force on Epidemiology and Prevention (National Heart, Lung, and Blood Institute; American Heart Association; World Heart Federation; International Atherosclerosis Society; and International Association for the Study of Obesity) [10] when three or more of the five conditions listed below were present (this definition recognizes that the risk associated with a particular waist measurement differs in different populations):

- Abdominal obesity defined as waist circumference > 102 cm in men and > 88 cm in women.
- Triglycerides ≥ 150 mg/dl.
- HDL cholesterol < 40 mg/dl for men and < 50 mg/dl for women.
- Blood pressure ≥ 130/ ≥ 85 mmHg.
- Fasting glucose ≥ 100 mg/dl (this condition was satisfied in patients with diabetes mellitus by definition).

Hypertension was defined as a resting blood pressure greater than 140 mmHg systolic and/or greater than 90 mmHg diastolic on at least two occasions or current antihypertensive pharmacological treatment. Obesity was recognized when body mass index ≥ 30 kg/m^2^. Dyslipidemia was defined as levels of total serum cholesterol > 190 mg/dl and or triglycerides > 150 mg/dl or pharmacologically treated high lipid serum levels. Fasting plasma glucose level of 7.0 mmol/l (126 mg/dl) or greater or treatment with oral hypoglycemic agent and/or insulin identified patients with diabetes mellitus. Renal function was assessed calculating the glomerular filtration rate (GFR) estimated by the CKD-EPI equation. Ischemic stroke was defined as a focal neurological deficit of sudden onset as diagnosed by a neurologist, lasting more than 24 h and caused by ischemia; transient ischemic attack (TIA) was defined as a focal neurological deficit of sudden onset and diagnosed by a neurologist, lasting less than 24 h; thromboembolism (TE) was defined as the occlusion of blood flow by an embolus, outside the brain and heart by the responsible physician. To stratify individuals according to the magnitude of risk for CV adverse clinical events, we used the “Italian Progetto CUORE risk score” which was built specifically for the Italian population miming the Framingham experience using data from different cohorts enrolled in the north, center and south of Italy between the 1980s and the 1990s, whose risk factors had been collected using standardized procedures [27]. A score > 2.5% identified subjects at moderate/high CV risk. The Charlson index was calculated in each patient to evaluate the degree of comorbidity/frailty [28]. We defined patients as biologic disease-modifying anti-rheumatic drugs (DMARDs) refractory on the date they had started their third class of biologic DMARDs before the enrollment into the study [29]. The degree of disease activity was evaluated by the clinical disease activity index (CDAI) score [30]. Patients with a CDAI score > 10 were defined as subjects with activated pattern of the disease having moderate-high disease activity.

### Echocardiography

Transthoracic Doppler echocardiography was performed following a standardized protocol. LV mass was calculated using the Devereux’s formula and normalized for height to the 2.7 power; LV hypertrophy was defined as LV mass > 49.2 g/m^2.7^ for men and > 46.7 for women [31]. LV end-diastolic and end-systolic volumes were measured by the biplane method of disks from 2D apical 4 chamber + 2 chamber views and used to calculate ejection fraction. Assessment of LV diastolic function was based on widely accepted diastolic function parameters and LV diastolic dysfunction was recognized using validated cutoffs of prognostic relevance, as previously reported [32].

### Outcomes and follow-up

The pre-specified primary end point of the study was a composite of all-cause death/all-cause hospitalization. Co-primary end point was a composite of CV death/CV hospitalization due to both cardiac events (unstable angina, myocardial infarction, severe chest pain due to acute pericarditis, heart failure, percutaneous coronary intervention and coronary artery bypass grafting) and vascular events (stroke, TIA, TE, peripheral vascular intervention and stent thrombosis). For each patient, the follow-up was stopped at the time of the first (CV or non-CV) event. All clinical events were examined by an independent end-point classification committee. Each clinical event was diagnosed and classified by two expert clinicians who analyzed in detail the clinical reports, validated the end points and formally generated the information which migrated into the database. Hospitalizations and vital status were recorded every 3 months during the scheduled visited for clinical check or during hospital access for therapy with biologic DMARDs or by telephone calls. Follow-up ended on 30 April 2019. All anamnestic data and those gathered during follow-up were recorded in the patient’s e-chart and then subsequently migrated to the data warehouse.

### Statistical analysis

Data are reported as mean values ± 1 standard deviation (medians and interquartile ranges for variables deviating from normality) or percentages. Unpaired Student’s test and χ^2^ statistics were used for descriptive statistics. Between-group comparisons of categorical and continuous variables were performed by χ^2^ test and analysis of variance (ANOVA) with comparison between each group by Scheffè test for unequal sample, as appropriate or the Mann–Whitney non-parametrical test. Multivariate logistic regression analysis was performed to assess the factors independently associated with MetS. Log cumulative hazard functions were computed by univariate and multivariate Cox regressions to identify the factors independently associated with the study clinical end points. Variables that were significantly related to the study end point in univariate tests (*p* ≤ 0.05) were included in the multivariable models, which also comprised those variables that were forced into the models for their specific clinical relevance. Probabilities of event-free survival and Kaplan–Meier survival curves of patients with vs those without MetS were obtained (differences between the curves were tested for significance by the Log-rank test). All analyses were performed using statistical package SPSS 19.0 (SPSS Inc. Chicago. Illinois, USA) and statistical significance was identified by two-tailed *p* < 0.05.

## Results

### Study population

The initial study population comprised 468 subjects. Among these patients, 10 (2%) were lost to the follow-up leaving 458 patients (228 RA, 96 AS, 134 PsA) who had complete clinical and follow-up data and formed the final population of the present study. Their baseline characteristics are shown in Table 1. Patients had a mean age of 58 ± 12 years, 63% were women, 16% obese. MetS was recognized in 73 subjects (15.6%). Prevalence of MetS was similar between patients with RA (34 of 228 = 15%), PsA (25 of 134 = 19%) and AS (14 of 96 = 15%; all *p* between the groups > 0.1).

**Table 1:** Baseline characteristics of the study population divided into two subgroups according to the presence/absence of metabolic syndrome

Variables	MetS No 385 patients	MetS Yes 73 patients	*p*	Total study population 458 patients
Age (years)	57 ± 13	62 ± 11	< 0.001	58 ± 12
Female gender (%)	63	61	0.66	63
Body mass index (Kg/height^2^)	25.3 ± 4.0	29.4 ± 5.3	< 0.001	25.9 ± 4.4
Waist circumference (cm)	90.9 ± 11.8	104.1 ± 12.2	< 0.001	93.0 ± 12.8
Obese (%)	12	39	< 0.001	16
Systolic blood pressure (mmHg)	129 ± 16	145 ± 18	< 0.001	131 ± 17
Diastolic blood pressure (mmHg)	82 ± 8	87 ± 9	0.04	82 ± 8
Hypertension (%)	40	80	< 0.001	46
Smoking (%)	34	34	0.99	34
Dyslipidemia (%)	54	68	0.03	57
Diabetes mellitus (%)	5	30	< 0.001	9
eGFR (ml/min/m^2^*1.73)	94 ± 22	93 ± 28	0.58	94 ± 23
Hemoglobin (g/dl)	13.9 ± 1.4	13.9 ± 1.6	0.80	13.9 ± 1.4
Glycemia (mg/dl)	89.9 ± 15.4	116.1 ± 29.2	< 0.001	95 ± 25
Cholesterol HDL (mg/dl)	65 [48–82]	52 [32–68]	< 0.001	61 [49–73]
Cholesterol LDL (mg/dl)	123 [93–151]	120 [85–138]	0.58	121 [99–140]
Triglycerides (mg/dl)	106 [72–134]	182 [115–245]	< 0.001	101 [74–139]
Progetto Cuore risk score (%)^a^	5.1 ± 3.9	12.2 ± 10.7	< 0.001	6.3 ± 5.2
Moderate/high CV risk (%)	57	82	< 0.001	61
Cancer (%)^b^	6	31	< 0.001	10
Charlson index (points)	2.4 ± 1.8	5.8 ± 2.4	< 0.001	2.9 ± 2.2
C-reactive protein (mg/dl)	4.0 [2.5–7.1]	6.2 [3.8–9.9]	0.04	4.3 [2.8–7.8]
ESR (mm/h)	19 [6, 29]	22 [11, 34]	0.22	15 [6, 27]
Rheumatoid factor positive (%)^c^	51	47	0.48	50
ACPA positive (%)^c^	50	46	0.28	49
Duration of disease (years)	12.7 ± 9.8	12.2 ± 10.1	0.67	12.6 ± 9.9
CDAI^c^	9.7 ± 8.9	13.7 ± 10.6	0.009	10.5 ± 8.4
Moderate/high disease activity (%)	28	48	< 0.001	32
LV mass (g/height^2.7^)	43 ± 11	48 ± 12	< 0.001	44 ± 11
E/E′ ratio	6.2 ± 1.5	7.1 ± 2.3	< 0.001	6.3 ± 1.7
LV diastolic dysfunction (%)	26	39	0.35	28
LVEF (%)	66 ± 6	66 ± 6	0.35	66 ± 6
Medications				
ACEi/ARBs (%)	23	56	< 0.001	28
Beta-blockers (%)	13	33	< 0.001	16
Diuretics (%)	13	27	0.002	15
Calcium antagonists (%)	9	10	0.67	9
Statins (%)	17	39	< 0.001	21
Anti-platelet agents (*n*, %)	11	22	< 0.001	13
NSAIDs (%)	36	30	0.38	35
Methotrexate (%)	40	46	0.34	41
Hydroxychloroquine (%)	8	6	0.69	8
Corticosteroids (%)	36	30	< 0.001	35
Biologic DMARDs at enrollment (%)	69	57	< 0.001	67
Biologic DMARDs class				
Anti-TNFα (%)^d^ Anti-interleukin 6 (%)^d^ CTLA 4Ig (%)^d^ Anti-CD 20 (%)^d^	70 12 12 6	64 11 16 9	0.10	69 12 13 6
Biologic DMARDs refractory (%)	28	30	0.79	28

*ACEi* angiotensin-converting enzyme inhibitors, *ACPA* anti-cyclic citrullinated peptide antibodies, *ARB* angiotensin T1 receptor blockers, *CDAI* clinical disease activity index, *CD* cluster of differentiation, *CTLA* cytotoxic T-lymphocyte antigen, *DMARDs* disease-modifying anti-rheumatic drugs, *ESR* erythrocyte sedimentation rate, *LVEF* left ventricular ejection fraction, *NSAIDs* non-steroidal anti-inflammatory drugs, *TNF* tissue necrosis factor

^a^Age, systolic blood pressure, total cholesterol, HDL-cholesterol, smoking habit, diabetes and hypertension treatment were included in the function; the first major coronary or cerebrovascular event was considered as end point; 10-year survival was assessed both for men and women

^b^% Included patients in whom cancer was diagnosed during follow-up

^c^% Among patients with rheumatoid arthritis

^d^% Among patients who were receiving biologic DMARDs

### MetS and study groups

The baseline clinical features of the 73 patients who had MetS were compared with those of 385 patients who had not (Table 1). The former were older, with a higher prevalence of all CV risk factors (but smoking and renal dysfunction), cancer, higher markers and scores of disease activity, and higher LV mass than the latter. Furthermore, patients with MetS were taking at enrollment more frequently drugs for the control of the CV risk factors, more frequently corticosteroids and biologic DMARDs than patients without MetS. Prevalence of biologic DMARDs refractoriness was similar between the two groups.

### Covariates of MetS

Variables significantly associated with MetS at univariate analysis are listed in the Table 2. Among this variables, age, cancer, C-reactive protein, moderate/high disease activity, LV mass and E/E′ ratio (parameter of LV diastolic function) were included in the multivariate logistic regression model. This analysis revealed that cancer, presence of moderate/high disease activity, higher LV mass and higher E/E′ ratio (index of higher degree of LV diastolic dysfunction) were the states independently associated with MetS in our patients.

**Table 2:** Variables significantly associated with metabolic syndrome: univariate and multivariate logistic regression analysis

Variables	Univariate analysis			Multivariate analysis		
	Odds ratio	Confidence intervals	*p*	Odds ratio	Confidence intervals	*p*
Age (years)	1.04	1.01–1.07	0.002	0.99	0.96–1.03	0.74
Cancer (%)^a^	7.45	3.84–14.44	< 0.001	4.78	2.19–10.41	< 0.001
C-reactive protein (mg/dl)	1.03	1.00–1.06	0.04	1.01	0.97–1.05	0.73
CDAI (%)	1.04	1.01–1.08	0.01			
Moderate/high disease activity (%)	2.28	1.29–4.03	0.004	1.77	1.01–3.27	0.04
LV mass (g/height^2.7^)	1.04	1.02–1.06	< 0.001	1.03	1.00–1.05	0.03
E/E′ ratio	1.33	1.13–1.52	< 0.001	1.19	1.02–1.40	0.04
LV diastolic dysfunction (%)	1.78	1.05–3.04	0.03			

^a^% Included patients in whom cancer was diagnosed during follow-up

### Clinical outcomes

Data on vital status and hospitalizations were available for all 458 patients. During a median follow-up of 36 months (IQR 23–45), eight patients (1.7%) died. All of them died during hospitalization. Causes of death were cancer in four patients (pancreatic in 2 cases, breast and laryngeal in one case), and congestive heart failure, complications of pneumonia, femoral neck fracture and peripheral vascular surgery in the remaining four patients.

A primary end point (all-cause death/all-cause hospitalization) occurred in 128 patients (28%). A co-primary end point (CV death/CV hospitalization) occurred in 37 patients (8%). Considering the primary end point, the event rate at 36-month follow-up was 52% in the group with MetS (38 of 73 patients) vs 23% in the group without MetS (90 of 385 patients, *p* < 0.001). In regard to the co-primary end point CV death/CV hospitalization, the event rate was significantly higher in the former group (11 patients = 15%) than in the latter group (26 patients = 7%, *p* = 0.02), as well as for non-CV events (38% vs 16%, *p* < 0.001).

Reasons of the 128 all-cause hospitalizations, separated in 37 CV hospitalizations and 91 non-CV hospitalizations, are reported in Table 3. Causes of CV hospitalization did not substantially differ between patients with and without MetS. Among the causes of non-CV hospitalization, cancer (9 patients = 12.7% vs 2 patients = 0.5%, *p* < 0.001), bone fracture (5 patients = 7.0% vs 11 patients = 2.8%, *p* = 0.03), and hip/knee arthroplasty (4 patients = 5.6% vs 11 patients = 2.8%, *p* = 0.04) occurred more frequently in patients with than without MetS, respectively. No significant difference was recognized between the two groups for the other causes of non-CV hospitalization.

**Table 3:** Causes of hospitalization during the follow-up

Cardiovascular hospitalization *N* = 37	MetS No 385 pts	MetS Yes 73 pts	Total population 458 pts
Number of events	26 (6.7%)	11 (15.1%)	37
Myocardial infarction, *n* (%)	2 (0.5)	2 (2.7)	4
Unstable angina (without any invasive procedure), *n* (%)	3 (0.8)	–	3
Percutaneous coronary intervention, *n* (%)	2 (0.5)	–	2
Coronary artery bypass grafting, *n* (%)	1 (0.2)	–	1
Acute congestive heart failure, *n* (%)	5 (1.3)	1 (1.4)	6
Chest pain due to acute pericarditis, *n* (%)	2 (0.5)	2 (2.7)	4
Stroke, *n* (%)	6 (1.6)	–	6
Atrial fibrillation (with hemodynamic instability), *n* (%)	4 (1.0)	3 (4.1)	7
Percutaneous peripheral artery intervention, *n* (%)	1 (0.2)	2 (2.7)	3
Acute peripheral ischemia (requiring amputation), *n* (%)	–	1 (1.4)	1
Non-cardiovascular hospitalization *N* = 91			
Number of events	64 (16.6%)	27 (37.0%)	91
Bone fracture	11 (2.8)	5 (6.8)	16
Hip or knee arthroplasty	11 (2.8)	4 (5.5)	15
Shoulder surgery	4 (1.0)	1 (1.4)	5
Joint arthrodesis	4 (1.0)	–	4
Joint synovectomy	4 (1.0)	1 (1.4)	5
Surgery for tendon rupture	2 (0.5)	–	2
Pneumonia	6 (1.5)	2 (2.7)	8
Non-pulmonary infection	7 (1.8)	3 (4.1)	10
Acute ulcerative rectocolitis	4 (1.0)	1 (1.4)	5
Autoimmune acute uveitis	5 (1.3)	1 (1.4)	6
Cancer	2 (0.5)	9 (12.3)	11
Thyroidectomy (no thyroid malignancy)	2 (0.5)	–	2
Acute pancreatitis	1 (0.2)	–	1
Dress syndrome	1 (0.2)	–	1

### MetS as prognosticator of adverse outcome

At univariate Cox regression, the variables associated with the primary study end point were hypertension, waist circumference, MetS, LV mass, duration of chronic inflammatory disease and biologic DMARDs refractoriness. All these variables together with age, which was forced into the model, were considered in the multivariate model. Multivariate Cox regression analysis revealed that MetS was independently related to the primary end point (HR 1.52 [CI 1.01–2.47], *p* = 0.04), together with higher LV mass, longer duration of chronic inflammatory disease and higher prevalence of biologic DMARDs refractoriness (Table 4, upper part).

**Table 4:** Variables associated with the study end points: univariate and multivariable Cox regression analyses

	Endpoint Yes	Endpoint No	Univariate				Multivariate		
			HR		CI	*p*	HR	CI	*p*
All-cause death/hospitalization	128 pts	330 pts							
Age (years)	61 ± 12	56 ± 13	1.01	0.99–1.02		0.34	1.01	0.98–1.03	0.52
Hypertension (%)	59	42	1.58	1.11–2.25		0.01	1.08	0.67–1.76	0.75
Dyslipidemia (%)	67	52	1.30	0.90–1.90		0.16			
Waist circumference (cm)	96 ± 12	91 ± 13	1.02	1.01–1.04		0.001	1.01	0.99–1.03	0.49
Metabolic syndrome (%)	52	23	1.80	1.20–2.68		0.003	1.52	1.01–2.47	0.04
E/E′	6.7 ± 1.9	6.1 ± 1.6	1.00	0.92–1.10		0.91			
Left ventricular mass (g/height^2.7^)	47 ± 11	43 ± 11	1.01	1.00–1.03		0.04	1.02	1.00–1.05	0.02
Disease duration (years)	15 ± 9	11 ± 7	1.02	1.00–1.04		0.04	1.02	1.00–1.04	0.02
Biologic DMARDs refractory (%)	39	23	1.71	1.14–2.58		0.01	1.66	1.07–2.57	0.03
Cardiovascular death/hospitalization	37 pts	421pts							
Age (years)	67 ± 11	57 ± 12	1.06	1.03–1.09		< 0.001	1.02	1.00–1.04	0.04
Hypertension (%)	62	45	1.90	0.98–3.70		0.06			
Dyslipidemia (%)	72	55	1.92	0.92–3.99		0.07			
Metabolic syndrome (%)	15	7	2.14	1.12–4.68		0.01	2.05	1.16–3.60	0.01
Glomerular filtration rate (ml/min/m^2^*1.73)	83 ± 20	95 ± 23	0.98	0.96–0.99		0.02	1.00	0.99–1.01	0.76
E/E′	7.7 ± 2.5	6.2 ± 1.5	1.38	1.19–1.59		0.01	1.07	0.93–1.22	0.35
Left ventricular mass (g/height^2.7^)	53 ± 12	43 ± 11	1.04	1.02–1.06		< 0.001	1.03	1.00–1.06	0.02
Disease duration (years)	16 ± 9	12 ± 7	1.03	0.99–1.06		0.06			

**Fig. 1:**
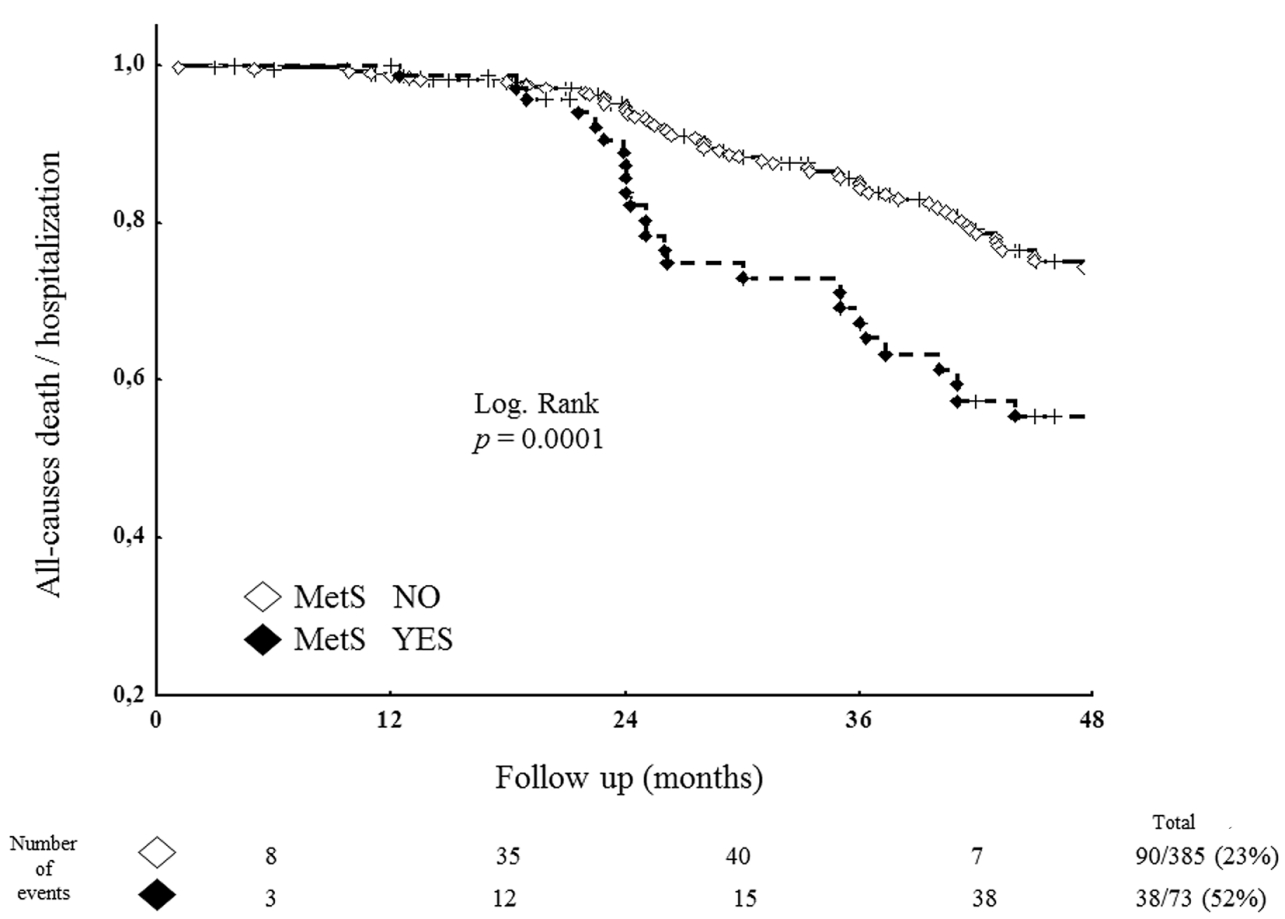
Kaplan–Meier survival curves from primary end point (all-cause death/hospitalization) of patients with metabolic syndrome (MetS) vs those without MetS. Total study population including 458 patients were analyzed

**Fig. 2:**
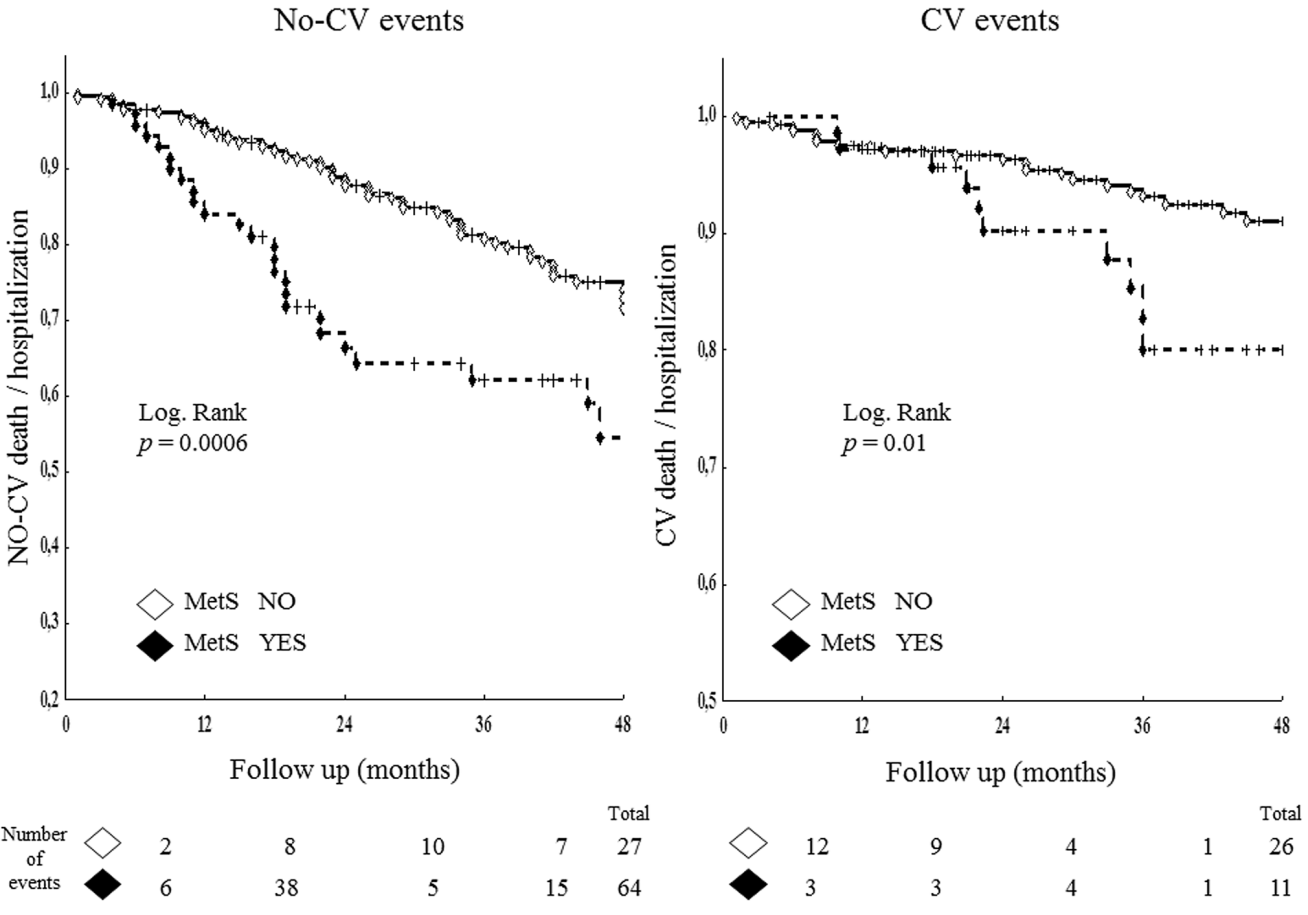
Kaplan–Meier survival curves from co-primary end point of patients with metabolic syndrome (MetS) vs those without MetS (right panel). Left panel refers to the non-CV events occurred in the two groups analyzed in the study

Considering the co-primary end point, the variables associated with the 37 adverse events at univariate Cox regression analysis were age, MetS, GFR, LV mass and E/E′, with hypertension, dyslipidemia and duration of chronic inflammatory disease, showing a borderline statistical significance. Multivariate Cox regression (including age, MetS, GFR, LV mas and E/E′) showed that MetS was independently related to CV death/CV hospitalization (HR 2.05 [CI 1.16–3.60], *p* = 0.01), together with older age and higher LV mass (Table 4, lower part). Kaplan–Meier survival curves for primary end point of patients with MetS vs those without MetS are shown in Fig. 1. Figure 2 shows Kaplan–Meier survival curves of the two study groups for co-primary end point (right panel) and for non-CV events analyzed separately (left panel).

## Discussion

Our study showed some original and clinically relevant results which have never been analyzed in depth in patients with chronic inflammatory arthritis: (1) MetS is recognized in a consistent proportion (around one-sixth) of subjects with RA/PsA/AS without overt cardiac disease selected for a primary prevention program for CV diseases; (2) in these patients, the presence of MetS is associated with cancer, presence of moderate/high disease activity, higher LV mass and E/E′ ratio (suggestive of higher degree of LV diastolic dysfunction) independently of the various components by which MetS is recognized; (3) MetS emerges by Cox regression analysis as a strong prognosticator of adverse clinical events at mid-term follow-up; (4) the relationship between MetS and poorer prognosis relates to both primary end point (all-cause death/all-cause hospitalization) and co-primary end point (CV death/CV hospitalization) and is independent of the traditional CV risk factors and to the markers/scores commonly used for grading the magnitude of inflammation.

MetS is a cluster of traditional risk factors including abdominal obesity, atherogenic dyslipidemia, hypertension, and insulin resistance [9, 10], all states having common metabolic pathways with chronic systemic inflammation. Thus, it is not surprising that the prevalence of MetS is significantly higher in patients with RA/PsA/AS as compared to the general population [22–24]. We demonstrated that RA/PsA/AS subjects with MetS have higher magnitude of inflammation and a fivefold risk of developing cancer compared with counterparts without MetS. Linear associations of single metabolic risk factors with risk of incident overall cancer has been found by Stocks et al. [33] and Nagel et al. [34] in pooled analyses of several community cohorts. Such associations were confirmed when a derived metabolic risk score of five metabolic factors was applied [35, 36]. However, chronic inflammatory arthritis predisposes to cancer [37] by the stimulation of B and T cells by various antigens activating pro-inflammatory/carcinogenic cytokynes and the use of immunosoppressive drugs [38, 39]. Collectively, our findings indicate that in patients with RA/PsA/AS, the relationship between higher magnitude of chronic inflammation and the risk of developing cancer is magnified by the presence of MetS.

Higher LV mass and degree of LV diastolic dysfunction are also conditions related to MetS in our patients as well as the development of cancer (as demonstrated in a recent study) [40]. In this view, it is well known that arterial hypertension, hyperglycemia, dyslipidemia and central obesity confer individually a clinically relevant pressure/volume overload on the CV system, an atherogenic and neuro-endocrine stimulus for excessive LV mass growth and alterations in LV diastolic properties. In subjects with MetS, these functional myocardial changes seem to result specifically from intrinsic cardiomyocyte alterations, irrespective of the myocardial interstitium (including fibrosis), as detected by cardiac magnetic resonance [41]. All together, these effects harmfully predispose to the development of subclinical structural and functional abnormalities of cardiac [11–15] and vascular function [16, 17], which herald subsequent adverse clinical events.

As a result, MetS is a powerful prognosticator for stroke and CV diseases [5, 11, 42–44], both in the general population [42, 43] and in some specific settings of patients such as those with arterial hypertension [12] and/or type 2 diabetes mellitus [44]. Furthermore, it has been associated with silent myocardial ischemia independent of essential hypertension [45]. However, exact information in regard to the prognostic role of MetS in patients with RA/AS/PsA is still lacking. In this study, we demonstrated that MetS is closely related to both all-cause and CV adverse events at mid-term, together with older age, longer disease duration, higher LV mass and biologic DMARDs refractoriness. In our experience, during 3 years of follow-up, an event leading to hospitalization for all causes or for CV cause occurred in more than half and one-sixth of our patients with MetS, respectively. Our results are in line with those of Rutter et al. [42], showing that MetS predicted CV events in the community population analyzed in the Framingham Offspring Study. Similar data emerged in the prospective study of Santaniemi et al. [5], who analyzed 1004 Finnish subjects showing after 18 years of observation a 2.01-fold higher probability for any CV event in individuals who had MetS compared with those who had not. The hazard ratio raised to 7.89 in the subgroup of patients in whom all five components of MetS were present.

It has been clearly demonstrated that the main metabolic alterations induced by MetS correlate with an increased production of some cytokines (i.e., TNF-α, IL-6), which interfere with adipocyte metabolism both in patients with and without chronic inflammatory arthritides [46, 47]. All these abnormalities lead to decreased rates of glucose oxidation and non-oxidative glucose disposal, high rates of lipid oxidation, adhesion molecules [48] and faster progression of atherosclerotic damage [49]. Although data on serum cytokines levels were not available, these conditions may possibly represent reasons why our patients with RA/AS/PsA and MetS have lower threshold of myocardial or arterial ischemia and higher CV event rate.

Of interest, besides the higher incidence of CV events, patients who had MetS showed an increased event rate for non-CV death/hospitalization (more than 2-fold higher) than those who had not. This finding suggests that MetS is not a mere expression of the pathophysiologic mechanisms associated with RA/AS/PsA disease and/or a CV risk factor, but an influential marker of frailty and an indicator of more advanced stage of inflammatory disease predisposing to adverse clinical events. The very large difference in the Charlson comorbidity index between patients with and without MetS supports this concept.

### Study limitations and strengths

Although prospective, our data were collected by a single center, so that some selection bias may have influenced the selection of patients. Secondly, the results of the present study might be prejudiced by the relatively small number of patients and/or CV events. Otherwise, the study strengths consist of the complete nature of the data set, the prospective gathering of quite a lot of variables traditionally related to CV and non-CV events, the use of a clinical, simple, feasible and validated definition on MetS and the accessibility to all prognostic information.

## Conclusions

Chronic inflammatory arthritis including RA, AS and PsA are conditions at increased risk of morbidity and mortality for which a prognostic assessment for supporting an effective clinical management is mandatory. MetS is frequent in these patients, being closely and directly related to chronic inflammation and disease activity. It is associated with cancer, presence of moderate/high disease activity, LV structural and functional abnormalities, and is an independent prognosticator of adverse clinical events at mid-term follow-up. In light of our results, an increasingly accurate assessment of MetS should be routinely conducted in patients with RA/AS/PsA as a measure of clinical outcomes which goes beyond the role of simple CV risk factor. Beside this, prospective investigations aimed to assess the potential favorable role of anti-IL-6/anti-TNF-α biologic DMARDs on the development and/or on the detrimental effect of MetS would be appropriate.

## References

[1] Lakka HM, Laaksonen DE, Lakka TA, Niskanen LK, Kumpusalo E, Tuomilehto J, Salonen JT (2002). The metabolic syndrome and total and cardiovascular disease mortality in middle-aged men. JAMA.

[2] Malik S, Wong ND, Franklin SS, Kamath TV, L’Italien GJ, Pio JR, Williams GR (2004). Impact of the metabolic syndrome on mortality from coronary heart disease, cardiovascular disease, and all causes in United States adults. Circulation.

[3] Schillaci G, Pirro M, Vaudo G, Gemelli F, Marchesi S, Porcellati C, Mannarino E (2004). Prognostic value of the metabolic syndrome in essential hypertension. J Am Coll Cardiol.

[4] Grassi G, Quarti-Trevano F, Seravalle G, Dell’Oro R (2007). Cardiovascular risk and adrenergic overdrive in the metabolic syndrome. Nutr Metab Cardiovasc Dis.

[5] Santaniemi M, Ukkola O, Malo E, Bloigu R, Kesäniemi YA (2014). Metabolic syndrome in the prediction of cardiovascular events: the potential additive role of hsCRP and adiponectin. Eur J Prev Cardiol.

[6] Kahn R, Buse J, Ferrannini E, Stern M (2005). The metabolic syndrome: time for a critical appraisal: joint statement from the American Diabetes Association and the European Association for the Study of Diabetes. Diabetes Care.

[7] Reilly MP, Rader DJ (2003). The metabolic syndrome: more than the sum of its parts?. Circulation.

[8] Ahmadi A, Leipsic J, Feuchtner G, Gransar H, Kalra D, Heo R, Achenbach S, Andreini D, Al-Mallah M, Berman DS, Budoff M, Cademartiri F, Callister TQ, Chang HJ, Chinnaiyan K, Chow B, Cury RC, Delago A, Gomez MJ, Hadamitzky M, Hausleiter J, Hindoyan N, Kaufmann PA, Kim YJ, Lin F, Maffei E, Pontone G, Raff GL, Shaw LJ, Villines TC, Dunning A, Min JK (2015). Is metabolic syndrome predictive of prevalence, extent, and risk of coronary artery disease beyond its components? Results from the multinational coronary CT angiography evaluation for clinical outcome: an international multicenter registry (CONFIRM). PLoS ONE.

[9] de Simone G, Devereux RB, Chinali M, Best LG, Lee ET, Galloway JM, Resnick HE (2007). Prognostic impact of metabolic syndrome by different definitions in a population with high prevalence of obesity and diabetes: the strong heart study. Diabetes Care.

[10] Alberti KG, Eckel RH, Grundy SM, Zimmet PZ, Cleeman JI, Donato KA, Fruchart JC, James WP, Loria CM, Smith SC Jr, International Diabetes Federation Task Force on Epidemiology and Prevention, Hational Heart, Lung, and Blood Institute, American Heart Association, World Heart Federation, International Atherosclerosis Society, International Association for the Study of Obesity. Harmonizing the metabolic syndrome: a joint interim statement of the International Diabetes Federation Task Force on Epidemiology and Prevention, National Heart, Lung, and Blood Institute, American Heart Association, World Heart Federation, International Atherosclerosis Society, and International Association for the Study of Obesity (2009) Circulation 120:1640–1645 10.1161/CIRCULATIONAHA.109.192644 19805654

[11] Chinali M, Devereux RB, Howard BV, Roman MJ, Bella JN, Liu JE, Resnick HE, Lee ET, Best LG, de Simone G (2004). Comparison of cardiac structure and function in American Indians with and without the metabolic syndrome (the Strong Heart Study). Am J Cardiol.

[12] de Simone G, Devereux RB, Chinali M, Roman MJ, Lee ET, Resnick HE, Howard BV (2009). Metabolic syndrome and left ventricular hypertrophy in the prediction of cardiovascular events: the Strong Heart Study. Nutr Metab Cardiovasc Dis.

[13] Tadic M, Cuspidi C, Majstorovic A, Pencic B, Backovic S, Ivanovic B, Scepanovic R, Martinov J, Kocijancic V, Celic V (2014). Does the metabolic syndrome impact left-ventricular mechanics? A two-dimensional speckle tracking study. J Hypertens.

[14] Faganello G, Cioffi G, Faggiano P, Candido R, Tarantini L, De Feo S, Di Lenarda A, de Simone G (2015). Does metabolic syndrome worsen systolic dysfunction in diabetes? The shortwave study. Acta Diabetol.

[15] Tadic M, Ivanovic B, Kostic N, Simic D, Matic D, Celic V (2012). Metabolic syndrome and left ventricular function: is the number of criteria actually important?. Med Sci Monit.

[16] Mule G, Cottone S, Mongiovi R, Cusimano P, Mezzatesta G, Seddio G, Volpe V, Nardi E, Andronico G, Piazza G, Cerasola G (2006). Influence of the metabolic syndrome on aortic stiffness in never treated hypertensive patients. Nutr Metab Cardiovasc Dis.

[17] Iannuzzi A, De Michele M, Bond MG, Sacchetti L, Fortunato G, Salvatore F, Mattiello A, Panico S, Rubba P (2005). Carotid artery remodeling in middle-aged women with the metabolic syndrome (from the “Progetto ATENA” study). Am J Cardiol.

[18] Medina G, Vera-Lastra O, Peralta-Amaro AL, Jiménez-Arellano MP, Saavedra MA, Cruz-Domínguez MP, Jara LJ (2018). Metabolic syndrome, autoimmunity and rheumatic diseases. Pharmacol Res.

[19] Dolcino M, Pelosi A, Fiore PF, Patuzzo G, Tinazzi E, Lunardi C, Puccetti A (2018). Long non-coding RNAs play a role in the pathogenesis of psoriatic arthritis by regulating microRNAs and genes involved in inflammation and metabolic syndrome. Front Immunol.

[20] Bartlett DB, Connelly MA, AbouAssi H, Bateman LA, Tune KN, Huebner JL, Kraus VB, Winegar DA, Otvos JD, Kraus WE, Huffman KM (2016). A novel inflammatory biomarker, GlycA, associates with disease activity in rheumatoid arthritis and cardio-metabolic risk in BMI-matched controls. Arthritis Res Ther.

[21] Maruotti N, d'Onofrio F, Cantatore FP (2015). Metabolic syndrome and chronic arthritis: effects of anti-TNF-α therapy. Clin Exp Med.

[22] Chung CP, Oeser A, Solus JF, Avalos I, Gebretsadik T, Shintani A (2008). Prevalence of the metabolic syndrome is increased in rheumatoid arthritis and is associated with coronary atherosclerosis. Atherosclerosis.

[23] Chen YJ, Wu CY, Shen JL, Chu SY, Chen CK, Chang YT (2008). Psoriasis independently associated with hyperleptinemia contributing to metabolic syndrome. Arch Dermatol.

[24] Malesci D, Niglio A, Mennillo GA, Buono R, Valentini G, La Montagna G (2007). High prevalence of metabolic syndrome in patients with ankylosing spondylitis. Clin Rheumatol.

[25] Aletaha D, Neogi T, Silman AJ, Funovits J, Felson DT, Bingham CO (2010). 2010 Rheumatoid arthritis classification criteria: an American College of Rheumatology/European League Against Rheumatism collaborative initiative. Arthritis Rheum.

[26] Rudwaleit M, Taylor WJ (2010). Classification criteria for psoriatic arthritis and ankylosing spondylitis/axial spondyloarthritis. Best Pract Res Clin Rheumatol.

[27] Palmieri L, Panico S, Vanuzzo D, Ferrario M, Pilotto L, Sega R, Gruppo di Ricerca del Progetto CUORE (2004). Evaluation of the global cardiovascular absolute risk: the Progetto CUORE individual score. Ann Ist Super Sanita.

[28] Charlson ME, Pompei P, Ales KL, MacKenzie CR (1987). A new method of classifying prognostic comorbidity in longitudinal studies: development and validation. J Chronic Dis.

[29] Kearsley-Fleet L, Davies R, De Cock D, Watson KD, Lunt M, Buch MH, BSRBR-RA Contributors Group; BSRBR-RA Contributors Group (2018). Biologic refractory disease in rheumatoid arthritis: results from the British Society for Rheumatology Biologics Register for Rheumatoid Arthritis. Ann Rheum Dis.

[30] Dhaon P, Das SK, Srivastava R, Dhakad U (2018). Performances of clinical disease activity index (CDAI) and simplified disease activity index (SDAI) appear to be better than the gold standard disease assessment score (DAS-28-CRP) to assess rheumatoid arthritis patients. Int J Rheum Dis.

[31] de Simone G, Devereux RB, Daniels SR, Koren MJ, Meyer RA, Laragh JH (1995). Effect of growth on variability of left ventricular mass: assessment of allometric signals in adults and children and their capacity to predict cardiovascular risk. J Am Coll Cardiol.

[32] Nagueh SF, Smiseth OA, Appleton CP, Byrd BF, Dokainish H, Edvardsen T (2016). Recommendations for the evaluation of left ventricular diastolic function by echocardiography: an Update from the American Society of Echocardiography and the European Association of Cardiovascular Imaging. J Am Soc Echocardiogr.

[33] Stocks T, Bjørge T, Ulmer H, Manjer J, Häggström C, Nagel G, Engeland A, Johansen D, Hallmans G, Selmer R, Concin H, Tretli S, Jonsson H, Stattin P (2015). Metabolic risk score and cancer risk: pooler analysis of seven cohorts. Int J Epidemiol.

[34] Nagel G, Stocks T, Späth D, Hjartåker A, Lindkvist B, Hallmans G, Jonsson H, Bjørge T, Manjer J, Häggström C, Engeland A, Ulmer H, Selmer R, Concin H, Stattin P, Schlenk RF (2012). Metabolic factors and blood cancers among 578,000 adults in the metabolic syndrome and cancer project (Me-Can). Ann Hematol.

[35] Esposito K, Chiodini P, Colao A, Lenzi A, Giugliano D (2012). Metabolic syndrome and risk of cancer: a systematic review and meta-analysis. Diabetes Care.

[36] Jinjuvadia R, Lohia P, Jinjuvadia C, Montoya S, Liangpunsakul S (2013). The association between metabolic syndrome and colorectal neoplasm: systemic review and meta-analysis. J Clin Gastroenterol.

[37] Turesson C, Matteson EL (2013). Malignancy as a comorbidity in rheumatic diseases. Rheumatology.

[38] Grivennikov SI, Greten FR, Karin M (2010). Immunity, inflammation, and cancer. Cell.

[39] Balkwill FR, Mantovani A (2012). Cancer-related inflammation: common themes and therapeutic opportunities. Semin Cancer Biol.

[40] Cioffi G, Viapiana O, Tarantini L, Ognibeni F, Orsolini G, Fassio A (2020). Cancer in adult patients with inflammatory arthritis is associated with high ascending aortic stiffness and left ventricular hypertrophy and diastolic dysfunction. Intern Emerg Med.

[41] Ladeiras-Lopes R, Moreira HT, Bettencourt N, Fontes-Carvalho R, Sampaio F, Ambale-Venkatesh B, Wu C, Liu K, Bertoni AG, Ouyang P, Bluemke DA, Lima JA (2018). Metabolic syndrome is associated with impaired diastolic function independently of MRI-derived myocardial extracellular volume: the MESA study. Diabetes.

[42] Rutter MK, Meigs JB, Sullivan LM, D’Agostino RB, Wilson PW (2004). C-reactive protein, the metabolic syndrome and prediction of cardiovascular events in the Framingham Offsprings Study. Circulation.

[43] Alexander CM, Landsman PB, Teutsch SM, Haffner SM, Third National Health and Nutrition Examination Survey (NHANES III) and National Cholesterol Education Program (NCEP) (2003). NCEP-defined metabolic syndrome, diabetes, and prevalence of coronary heart disease among NHANES III participants age 50 years and older. Diabetes.

[44] Yang GR, Yuan MX, Fu HJ, Wan G, Li D, Dye TD, Zhu LX, Xie RR, Lv YJ, Zhang JD, Du XP, Li YL, Ji Y, Li Y, Cui XL, Wang ZM, Cheng SY, Liu DY, Wang Q, Zhou L, Gao Y, Yuan SY (2019). The association between metabolic syndrome and morbid events in type 2 diabetes after a 7-year community management: Beijing Community Diabetes Study 17. J Diabetes Res.

[45] Rendina D, Ippolito R, De Filippo G, Muscariello R, De Palma D, De Bonis S (2017). Risk factors for silent myocardial ischemia in patients with well-controlled essential hypertension. Intern Emerg Med.

[46] Sethi JK, Hotamisligil GS (1999). The role of TNF alpha in adipocyte metabolism. Semin Cell Dev Biol.

[47] Svenson KL, Lithell H, Hällgren R, Selinus I, Vessby B (1987). Serum lipoprotein in active rheumatoid arthritis and other chronic inflammatory arthritides. I. Relativity to inflammatory activity. Arch Intern Med.

[48] Salmenniemi U, Ruotsalainen E, Pihlajamäki J, Vauhkonen I, Kainulainen S, Punnonen K, Vanninen E, Laakso M (2004). Multiple abnormalities in glucose and energy metabolism and coordinated changes in levels of adiponectin, cytokines, and adhesion molecules in subjects with metabolic syndrome. Circulation.

[49] Luna-Luna M, Medina-Urrutia A, Vargas-Alarcón G, Coss-Rovirosa F, Vargas-Barrón J, Pérez-Méndez Ó (2015). Adipose tissue in metabolic syndrome: onset and progression of atherosclerosis. Arch Med Res.

